# Supplementary material for: Triacylglycerol mobilization underpins mitochondrial stress recovery
Source: Nat Cell Biol. 2025 Jan 8;27(2):298–308. doi: 10.1038/s41556-024-01586-6 (PMC11821527; doi:10.1038/s41556-024-01586-6)
Supplement: Supplementary file 1 — Reporting Summary [file 41556_2024_1586_MOESM1_ESM.pdf]

nature portfolio

Corresponding author(s): David J. Pagliarini

Last updated by author(s): Nov 25, 2024

Reporting Summary

Nature Portfolio wishes to improve the reproducibility of the work that we publish. This form provides structure for consistency and transparency in reporting. For further information on Nature Portfolio policies, see our Editorial Policies and the Editorial Policy Checklist.

Statistics

For all statistical analyses, confirm that the following items are present in the figure legend, table legend, main text, or Methods section.

n/a	Confirmed	
☐	☒	The exact sample size (*n*) for each experimental group/condition, given as a discrete number and unit of measurement
☐	☒	A statement on whether measurements were taken from distinct samples or whether the same sample was measured repeatedly
☐	☒	The statistical test(s) used AND whether they are one- or two-sided<br>*Only common tests should be described solely by name; describe more complex techniques in the Methods section.*
☒	☐	A description of all covariates tested
☒	☐	A description of any assumptions or corrections, such as tests of normality and adjustment for multiple comparisons
☐	☒	A full description of the statistical parameters including central tendency (e.g. means) or other basic estimates (e.g. regression coefficient) AND variation (e.g. standard deviation) or associated estimates of uncertainty (e.g. confidence intervals)
☐	☒	For null hypothesis testing, the test statistic (e.g. *F*, *t*, *r*) with confidence intervals, effect sizes, degrees of freedom and *P* value noted<br>*Give P values as exact values whenever suitable.*
☒	☐	For Bayesian analysis, information on the choice of priors and Markov chain Monte Carlo settings
☒	☐	For hierarchical and complex designs, identification of the appropriate level for tests and full reporting of outcomes
☒	☐	Estimates of effect sizes (e.g. Cohen's *d*, Pearson's *r*), indicating how they were calculated

Our web collection on statistics for biologists contains articles on many of the points above.

Software and code

Policy information about availability of computer code

Data collection	Quantitative PCR data was collected using QuantStudio Real-Time PCR software v1.2 (Applied Biosciences).<br>Growth assay, OCR, Florescent data was collected using Gen5 v3.02.2 (BioTek).<br>GO term enrichments were determined using ShinyGO v0.80 (http://bioinformatics.sdstate.edu/go/)<br>Florescent Microscopy images were taken using Zenn v.3.6 (Zeiss)<br>Volume density imaging was acquired using Elements v5.21 (Nikon)
Data analysis	LC-MS targeted lipidomics was analyzed by Tracefinder 5.1 (Thermo)<br>LC-MS untargeted lipidomics was analyzed by Lipidex v1.0<br>LC_MS proteomic files were analyzed using either MaxQuant v1.5.5.5 or Proteome Discoverer v2.5 (Thermo)<br>LC-MS Metabolomics was analyzed using Compound Discoverer (v3.1 & v3.3) (Thermo)<br>Florescent images were analyzed using ImageJ software v2.9.01.53t<br>Volume density imaging was analyzed using Ilastik (v.1.3.3) and YeaZ (v1.0.3)<br>Statistical analysis and all graphing was performed using Python v3.9<br>Cell and organelle analysis was performed using custom code (Github: https://github.com/alinearra/CellandOrganelleAnalysis)

For manuscripts utilizing custom algorithms or software that are central to the research but not yet described in published literature, software must be made available to editors and reviewers. We strongly encourage code deposition in a community repository (e.g. GitHub). See the Nature Portfolio guidelines for submitting code & software for further information.

Data

Policy information about availability of data

All manuscripts must include a data availability statement. This statement should provide the following information, where applicable:

- Accession codes, unique identifiers, or web links for publicly available datasets
- A description of any restrictions on data availability
- For clinical datasets or third party data, please ensure that the statement adheres to our policy

All mass spectrometry data (proteomics, lipidomics, and metabolomics) have been deposited in Massive with the primary accession codes MSV000092267 (multiomic screen) and MSV000095028 (follow up experiments). Source data, including all mass spectrometry data tables, have been provided in Source Data. The following databases were used in the searching of mass spectrometry files (Biocyc (https://www.biocyc.org/), Human Metabolome Database (https://hmdb.ca/), KEGG (https://www.genome.jp/kegg/), mzCloud (https://www.mzcloud.org/), MassBank (https://massbank.eu/MassBank/), MitoCarta (https://personal.broadinstitute.org/scalvo/MitoCarta3.0/human.mitocarta3.0.html), Uniprot (https://www.uniprot.org/)). All other data supporting the findings of this study are available from the corresponding author on reasonable request.

Research involving human participants, their data, or biological material

Policy information about studies with human participants or human data. See also policy information about sex, gender (identity/presentation), and sexual orientation and race, ethnicity and racism.

Reporting on sex and gender	N/A
Reporting on race, ethnicity, or other socially relevant groupings	N/A
Population characteristics	N/A
Recruitment	N/A
Ethics oversight	N/A

Note that full information on the approval of the study protocol must also be provided in the manuscript.

Field-specific reporting

Please select the one below that is the best fit for your research. If you are not sure, read the appropriate sections before making your selection.

☒ Life sciences ☐ Behavioural & social sciences ☐ Ecological, evolutionary & environmental sciences

For a reference copy of the document with all sections, see nature.com/documents/nr-reporting-summary-flat.pdf

Life sciences study design

All studies must disclose on these points even when the disclosure is negative.

Sample size	No statistical methods were used to predetermine sample size. All experiments were performed in at least biological triplicate. Samples sizes were chosen based on the generally accepted standard for the minimum number of replicates needed to obtain conclusive evidence for these types of experiments reported in previous publications.
Data exclusions	No data were excluded from these analyses.
Replication	All attempts at experimental replication were successful. All experiments were performed in at least biological triplicate, as indicated in the figure legends.
Randomization	Randomization was used for mass spectrometry injection to minimize batch effects. No other randomization was used for experimental groups as all other measurements were quantitatively made by instruments or made using computational analyses as is standard and previously reported.
Blinding	Blinding of experimental groups was not relevant as experimental measurements were generated by automated measurements or computational analyses.

Reporting for specific materials, systems and methods

We require information from authors about some types of materials, experimental systems and methods used in many studies. Here, indicate whether each material, system or method listed is relevant to your study. If you are not sure if a list item applies to your research, read the appropriate section before selecting a response.

Materials & experimental systems

n/a	Involved in the study
☒	☐ Antibodies
☐	☒ Eukaryotic cell lines
☒	☐ Palaeontology and archaeology
☒	☐ Animals and other organisms
☒	☐ Clinical data
☒	☐ Dual use research of concern
☒	☐ Plants

Methods

n/a	Involved in the study
☒	☐ ChIP-seq
☒	☐ Flow cytometry
☒	☐ MRI-based neuroimaging

Eukaryotic cell lines

Policy information about cell lines and Sex and Gender in Research

Cell line source(s)	HAP1 wild type - Horizon Discovery C631<br>HAP1 CPT2 KO - Horizon Discovery HZGHC003795c011<br>HAP1 ATGL KO - Horizon Discovery HZGHC004666c014
Authentication	HAP1 cell lines were authenticated by Horizon Discovery using PCR amplification and Sanger sequencing.
Mycoplasma contamination	All cell lines were negative for mycoplasma contamination as tested using a commercial test kit.
Commonly misidentified lines (See ICLAC register)	No commonly misidentified cell lines were used in this study.

Plants

Seed stocks	N/A
Novel plant genotypes	N/A
Authentication	N/A
